# Synthetic mycobacterial diacyl trehaloses reveal differential recognition by human T cell receptors and the C-type lectin Mincle

**DOI:** 10.1038/s41598-021-81474-3

**Published:** 2021-01-21

**Authors:** Josephine F. Reijneveld, Mira Holzheimer, David C. Young, Kattya Lopez, Sara Suliman, Judith Jimenez, Roger Calderon, Leonid Lecca, Megan B. Murray, Eri Ishikawa, Sho Yamasaki, Adriaan J. Minnaard, D. Branch Moody, Ildiko Van Rhijn

**Affiliations:** 1 Division of Rheumatology, Inflammation, and Immunity, Brigham and Women’s Hospital and Harvard Medical School, Hale Building for Transformative Medicine, 60 Fenwood Road, Boston, MA 02115 USA; 2 Department of Infectious Diseases and Immunology, Faculty of Veterinary Medicine, Utrecht University, Utrecht, The Netherlands; 3 Stratingh Institute for Chemistry, University of Groningen, Groningen, The Netherlands; 4 Socios En Salud, Lima, Peru; 5 Division of Global Health Equity, Department of Global Health and Social Medicine, Brigham and Women’s Hospital and Harvard Medical School, Boston, MA USA; 6 Department of Molecular Immunology, Research Institute for Microbial Diseases, Osaka University, Suita, Osaka Japan; 7 Laboratory of Molecular Immunology, Immunology Frontier Research Center, Osaka University, Suita, Osaka Japan

**Keywords:** Immunology, Glycolipids

## Abstract

The cell wall of *Mycobacterium tuberculosis* is composed of diverse glycolipids which potentially interact with the human immune system. To overcome difficulties in obtaining pure compounds from bacterial extracts, we recently synthesized three forms of mycobacterial diacyltrehalose (DAT) that differ in their fatty acid composition, DAT_1_, DAT_2_, and DAT_3_. To study the potential recognition of DATs by human T cells, we treated the lipid-binding antigen presenting molecule CD1b with synthetic DATs and looked for T cells that bound the complex. DAT_1_- and DAT_2_-treated CD1b tetramers were recognized by T cells, but DAT_3_-treated CD1b tetramers were not. A T cell line derived using CD1b-DAT_2_ tetramers showed that there is no cross-reactivity between DATs in an IFN-γ release assay, suggesting that the chemical structure of the fatty acid at the 3-position determines recognition by T cells. In contrast with the lack of recognition of DAT_3_ by human T cells, DAT_3,_ but not DAT_1_ or DAT_2_, activates Mincle. Thus, we show that the mycobacterial lipid DAT can be both an antigen for T cells and an agonist for the innate Mincle receptor, and that small chemical differences determine recognition by different parts of the immune system.

## Introduction

Infection with *Mycobacterium tuberculosis* (Mtb) in humans elicits a T cell response. Detection of T cell responses to peptide antigens from Mtb, presented by major histocompatibility complex (MHC) proteins, forms the basis of the most reliable diagnostic assay for infection with Mtb^1^. In addition to peptide antigens, cell wall lipids of Mtb have been shown to be presented by MHC class I-like proteins CD1a, CD1b, CD1c, and CD1d to T cells^2–10^. The non-polymorphic nature of CD1 molecules makes lipids presented by CD1 proteins ideal targets for vaccine approaches and diagnostic purposes^11^.

Among the many cell wall lipids of Mtb, some are present in most actinomycetes, whereas others exist among mycobacteria only, and some are strictly specific for the species Mtb. Mycobacterial lipids, such as mannosyl phosphomycoketide^2,4^, phosphomycoketide^6^, glucose monomycolate^5^, mycolic acid^8^, glycerol monomycolate^7^, diacyl sulfoglycolipid (Ac_2_SGL)^9^, and dideoxymycobactin^3^ have been shown to induce T cell responses via presentation by CD1 proteins. Contrary to the broad cross-reactivity against self and bacterial phospholipids^12,13^, individual mycobacterial lipid-specific T cell clones show high specificity for the hydrophilic headgroup of Mtb lipids, while the hydrophobic parts of the lipid that are buried deep in the CD1 cleft are not typically recognized by the T cell receptor through direct contact^14^. Parts of the fatty acids that lie on the CD1 surface or sit near the antigen exit portal, might contribute to T cell recognition and specificity, especially when they show distinguishing features like double bonds, hydroxylations, and methylations.

Not all Mtb (glyco)lipids have been studied as T cell antigens. We propose that if a lipid binds sufficiently to a CD1 molecule, and it has clear features that distinguish it from common self-lipids like phospholipids and sphingolipids, it may be specifically recognized by T cells. Because CD1 interacts with lipid antigens via hydrophobic interactions, any glycolipid with one, two, or three hydrophobic tails and a suitable size might bind to CD1. One of these candidate lipids is diacyl trehalose (DAT). DAT is suggested to be part of the external surface of the mycobacterial cell wall^15^ and belongs to the family of trehalose-based glycolipids, which includes lipids such as Ac_2_SGL. Although known as a biological substance for decades, DAT had not been chemically synthesized until recently^16^.

Besides functioning as lipid antigens for T cells, some mycobacterial lipids induce an innate response through pattern recognition receptors, such as the family of C-type lectin receptors (CLRs). The macrophage inducible Ca^2+^-dependent lectin (Mincle) receptor is one of the human CLRs. Trehalose-6,6′-dimycolate, a highly abundant glycolipid in the mycobacterial cell wall, was the first known Mtb lipid to activate the murine and human Mincle receptor^17^. Since then several natural and synthetic mycobacterial lipids have been shown to act as agonists for both the murine and human Mincle receptor, including DAT isolated from Mtb^18^. There has been growing interest in using Mincle ligands as adjuvants to promote a Th1 and Th17 immune response to subunit vaccines^19,20^.

Here, we took advantage of precisely defined synthetic forms of DAT to discover receptor mediated human cellular responses. Three synthetic DATs were tested for their potential as Mincle and T cell receptor (TCR) ligands. We developed CD1b tetramers loaded with synthetic DAT to study recognition of DAT by T cells ex vivo in both healthy individuals and tuberculosis patients.

## Results

### Validation of synthetic diacyl trehalose

**Figure 1:**
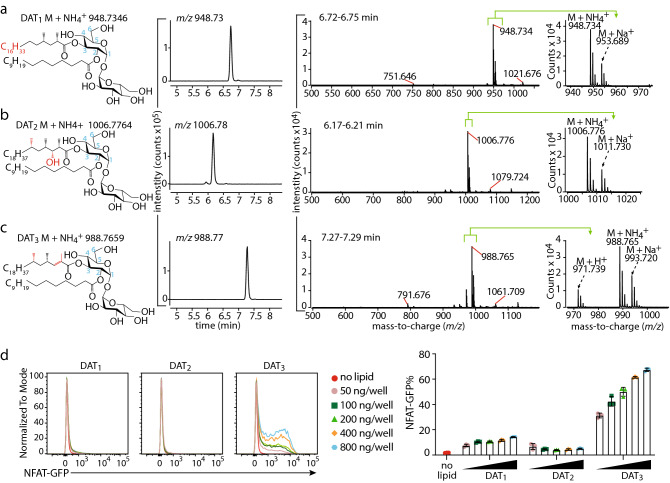
Synthetic DAT is a Mincle ligand. Synthetic DAT_1_ (**A**), DAT_2_ (**B**), and DAT_3_ (**C**) were analyzed by via high-performance liquid chromatography–mass spectroscopy (HPLC–MS). Extracted ion chromatograms were generated for the ammoniated molecular ions. The mass spectra at the elution time of the ammoniated ions contained the expected molecular ions with H^+^, NH_4_^+^, and Na^+^ adducts. Loss of hexose due to in-source fragmentation (calculated m/z 751.645 for DAT_1_ and 791.676 for DAT3) and sodium formate adduction with the Na+ adducted molecular ion (calculated m/z 1021.677 for DAT_1_, 1079.719 for DAT2, and 1061.708 for DAT3) accounted for additional small peaks. Differences between the three forms of DAT are indicated in red in the structural formula, and the numbering of the trehalose carbons is shown in blue. (**D**) NFAT-GFP reporter cells expressing mouse Mincle + FcRγ were stimulated with the indicated amount of DAT_1_, DAT_2_, DAT_3_, or TDM. After 24 h the induction of NFAT-GFP was analyzed by flow cytometry as shown in histogram and overlay flow cytometry histograms. Bar graphs represent three independent experiments. Values of each experiment are shown as symbols. Error bar represents standard deviation.

Natural DAT isolated from the cell wall of *M. tuberculosis* (Mtb) is a mixture of compounds that has immunomodulatory properties^21^. We recently synthesized three forms of DAT: DAT_1_, DAT_2_, and DAT_3_, that differ in the fatty acyl unit esterified to the 3-position of the glucose moiety in trehalose, where DAT_1_ carries mycosanoic acid, DAT_2_ carries mycolipanolic acid, and DAT_3_ carries mycolipenic acid^16^. Synthetic DAT_1_ and DAT_3_ were previously demonstrated to be identical to natural products, but synthetic DAT_2_ possessed identical fragmentation patterns to natural product, but did not co-elute by HPLC, suggesting that the two molecules are stereoisomers^16^. As a validation of the synthesized compound structure and quality after storage, high-performance liquid chromatography-mass spectrometry (HPLC–MS) analysis was performed (Fig. 1A–C). All three compounds yielded molecular ions with *m/z* values that, within experimental error, were consistent with the ammoniated synthesized target structures (*m/z* 948.735, 1006.776, and 988.766 for DAT_1_, DAT_2_, and DAT_3_ respectively). Each synthetic compound gave a single major chromatographic peak, consistent with isomeric purity. Retention times in the reversed phase method are expected to increase with molecular size but decrease with increasing polarity of groups, such as the hydroxy group on the hydrocarbon chain, and the relative retention times of the synthetic DATs matched this prediction as DAT_2_ was < DAT_1_, with DAT_3_ showing the longest retention time. Thus, the compounds showed high purity, correct mass and the expected retention times, allowing biological investigations of the antigenicity of DAT.

### Diacyl trehalose acts as a Mincle ligand

Both natural mixed and synthetic forms of DAT can be ligands for macrophage-inducible C-type lectin (Mincle), a receptor of the innate immune system and activator of macrophages that responds to several types of trehalose containing glycolipids^16,18^. To confirm that the stored synthetic lipids are bioactive, we tested their ability to activate Mincle by measuring the activation of NFAT-GFP in reporter cells expressing murine Mincle and its signaling subunit, the FcRγ chain. Synthetic DAT_3_ acts as an agonist for Mincle, while stimulation of Mincle by DAT_1_ and DAT_2_ was not much higher than background even at the highest concentrations tested (Figs. 1D and S1), consistent with the previously reported pattern^16^. These results confirm that the chemical structure of DAT influences recognition by Mincle and that DAT_3_ is a strong activator of Mincle.

### Identification of CD1b-DAT tetramer-specific T cells

**Figure 2:**
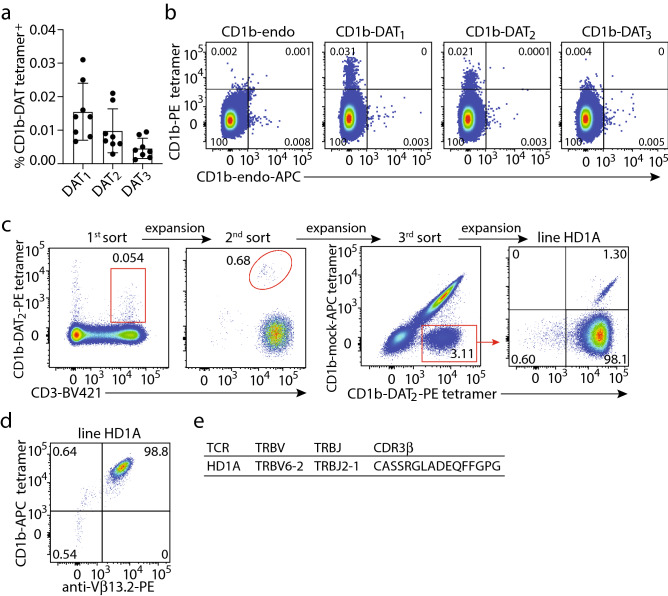
Identification of CD1b-DAT binding human T cells. (**A**) Percentages of CD1b-DAT_1_, -DAT_2_, and -DAT_3_ tetramer^+^ T cells of total T cells enriched from PBMC by column purification (n = 8 PBMC samples) are shown. (**B**) Flow cytometry dot plots show CD1b-endo, -DAT_1_, -DAT_2_, and -DAT_3_ staining on T cells from one of the healthy donors. (**C**) A T cell line was generated by sorting peripheral blood mononuclear cells from Healthy Donor 1 (HD1) based on expression of CD3 and binding to CD1b tetramers treated with DAT_2_ or mock treated (CD1b-mock), followed by expansion in vitro after each sort. Numbers next to or in the outlined red areas indicate percent cells in gate. (**D**) Flow cytometry dot plots show anti-CD1b-and anti-Vβ13.2 (TRBV6-2) staining on HD1A cell line. (**E**) TCR β chain sequence of HD1A TCR determined using a multiplex PCR and Sanger sequencing-based approach.

Regardless of their capacity to stimulate the innate immune system, it is possible that DAT_1_, DAT_2_, or DAT_3_ can function as foreign lipid antigens presented by CD1 proteins for human T cells. Among human CD1 proteins, CD1b can present lipids with the longest and most alkyl chains^22^. The ~ C42 forms of DAT studied here were somewhat larger than most lipids presenting by other CD1 isoforms, so we hypothesized that DAT could be presented by CD1b molecules to activate CD1b reactive T cells. To test this, we enriched T cells from healthy donor peripheral blood mononuclear cells (PBMCs) by depleting non-T cells using magnetic selection and stained them with CD1b tetramers that were treated with either synthetic DAT_1_, synthetic DAT_2_, or synthetic DAT_3_. Some T cells recognize 'CD1b-endo' complexes, which are so named because they carry endogenous self-phospholipids from the mammalian CD1 protein expression system. Such T cells recognize CD1b-phosholipid or bind the CD1b protein itself independent of the lipid bound^12,13^. Therefore, we stained the T cells simultaneously with a phycoerythrin (PE)-labeled synthetic DAT-treated CD1b tetramer and an allophycocyanin (APC)-labeled untreated CD1b tetramer (CD1b-endo). For quantification, residual non-T cells and auto-fluorescent cells were gated out, as well as CD1b-endo positive cells to determine true CD1b-DAT tetramer binding cells (Fig. S2a). CD1b-DAT tetramer^+^ cells were detected in all eight donors tested (Fig. 2A). Binding of CD1b-DAT_1_ tetramers was the highest, with frequencies ranging from 0.031 to 0.007% of total T cells, followed by CD1b-DAT_2_ (0.021–0.003%) and CD1b-DAT_3_, which was the lowest (0.009–0.001%). Visualization of double staining of T cells with CD1b-DAT- and CD1b-endo tetramers, not gating CD1b-endo tetramer^+^ cells out (Fig. S2b), showed that most CD1b-DAT tetramer-binding cells fail to bind CD1b-endo, as illustrated by dot plots from donor 49 (Fig. 2B) or the other donors (Fig. S3). Together these results suggest that synthetic DAT_1_ and synthetic DAT_2_ are T cell antigens, while we could not convincingly detect CD1b-synthetic DAT_3_ binding TCRs. Although we expect the three forms of DAT to load with comparable efficiency onto CD1b, we cannot formally exclude the possibility that DAT_3_ was less efficiently loaded. Therefore, our inability to detect CD1b-DAT_3_ binding T cells can be due to their absence in blood, or a failure to load tetramers with DAT_3_.

To enable functional studies of T cell response, we stained PBMC from a healthy blood bank donor (HD1) with anti-CD3 and CD1b-synthetic DAT_2_ tetramer. After two rounds of sorting and expansion of cells that were positive for CD3 and tetramer, we obtained an oligoclonal T cell line that, upon flow cytometric analysis, was demonstrated to consist mainly of T cells that stained double positive for mock treated CD1b (CD1b-mock) and CD1b-DAT_2_ tetramer. The approximately 3% of the cells in the cell line that stained brightly with CD1b-DAT_2_ tetramer but were negative for CD1b-mock tetramer were sorted and expanded further (Fig. 2C, third sort) to generate the 98% pure cell line HD1A (Fig. 2C, right panel). A 1.3% contamination of CD1b-mock^+^ cells was detected, which was not surprising because it formed the majority of the cells before the third sort. We further characterized line HD1A by staining with a panel of 24 Vβ antibodies (Fig. S4) and identified that it was an Vβ T cell line that stained with anti-Vβ13.2, which stains the TRBV6-2 gene product (Fig. 2D). Expression of TRBV6-2 was confirmed by a multiplex PCR approach (Fig. 2E). Thus, we were able to detect CD1b-DAT tetramer-binding T cells ex vivo and derived a TRBV6-2^+^ synthetic DAT_2_-specific αβT cell line.

### Primary CD1b-DAT recognizing T cells show functional responses to antigen

**Figure 3:**
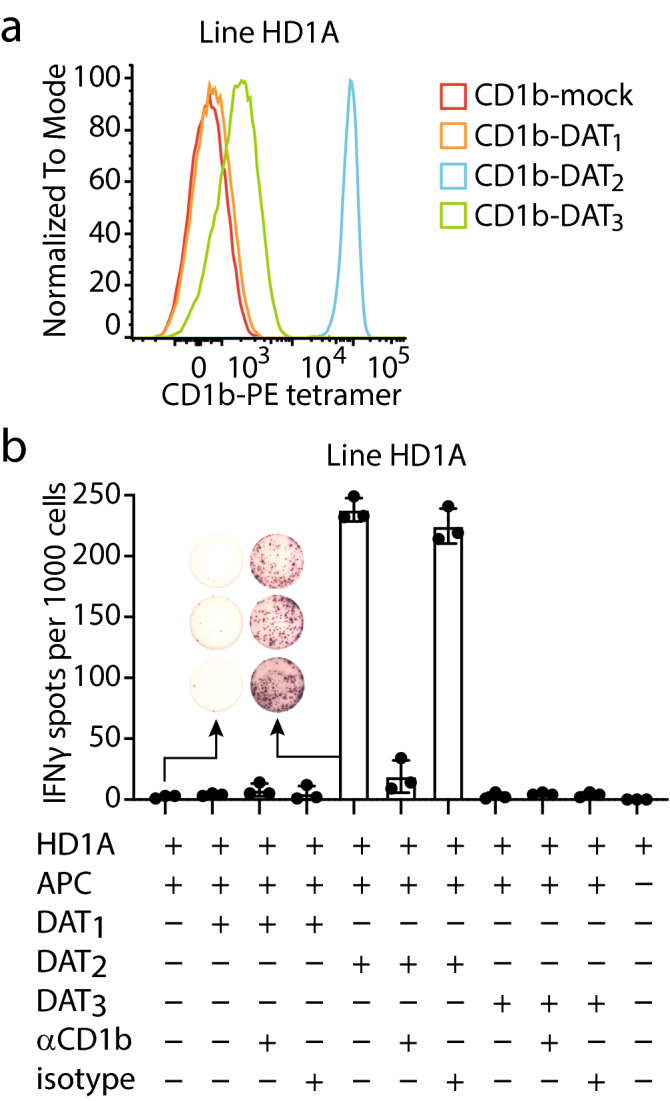
Line HD1A is reactive to DAT_2_. (**A**) Flow cytometry histogram of line HD1A stained with DAT_1_-, DAT_2_-, or DAT_3_-, or mock-treated CD1b tetramers (CD1b-mock). (**B**) IFN-γ ELISPOT of line HD1A stimulated with monocyte-derived dendritic cells treated with DAT_1_, DAT_2_, or DAT_3,_ with or without anti-CD1b antibody or Isotype control. Error bars represent standard error of the mean (SEM) of triplicate wells. One representative experiment of three is shown.

For some CD1b-presented lipid antigens, the exact composition of the lipid tail does not matter for T cell recognition^5^. However, for other CD1b antigens, such as Ac_2_SGL, mycolic acid, and mannosyl phosphomycoketide, the length and configuration of the acyl tail influences T cell activation^2,23–25^. For these three lipids it was shown that differences in the structure of the acyl tails, such as length of the acyl chain and the number or pattern of C-methyl branched groups changes recognition by the TCR. We wondered whether the diversity in the acyl chains of the three synthesized DATs could influence recognition by T cells. Therefore, we asked whether T cell line HD1A that was sorted with CD1b-synthetic DAT_2_ tetramers would be cross-reactive to the other synthetic DATs. Line HD1A, which stained strongly with CD1b tetramers treated with DAT_2_, did not stain with DAT_1_-treated tetramers more than the background obtained with mock-treated tetramers (Fig. 3A). CD1b-DAT_3_-treated tetramers showed a weak staining.

Tetramer staining suggests that the T cells would be responsive to the lipid antigen loaded onto the tetramers, but not all tetramer-binding T cells show functional responses upon presentation of antigen by antigen-presenting cells. To investigate functional responses to cellular presentation of synthetic DATs, we tested whether HD1A cells are functionally reactive to monocyte-derived dendritic cells, which represent in vitro generated primary APCs^26^, treated with synthetic DATs. DAT_2_ induced secretion of interferon-γ (IFN-γ) by HD1A T cells, while APCs treated with DAT_1_, DAT_3_ or medium alone, did not (Fig. 3B). Production of IFN-γ was almost completely blocked by anti-CD1b antibodies, indicating that CD1b is necessary for the activation of the HD1A by antigen, but not by other receptors present on the APCs, including CD1a, CD1c, or CD1d. Of note, synthetic DAT_3_, which supported low CD1b tetramer staining, did not lead to IFN-γ responses, which is most likely due to low potency of DAT_3_ as an antigen for HD1A. Thus, the HD1A cell line shows CD1b-dependent, highly specific functional responses to synthetic DAT_2_ presented by APCs, which is likely caused by TCR recognition of the CD1b-DAT_2_ complex. The lack of functional responses to DAT_1_ and DAT_3_ suggests that the chemical differences among DATs, consisting of the differing fatty acyl units at C3 of trehalose (Fig. 1A), influence recognition by T cells and prevents cross-reactivity.

### CD1b-DAT_2_ binding T cells in Peruvian cohort

**Figure 4:**
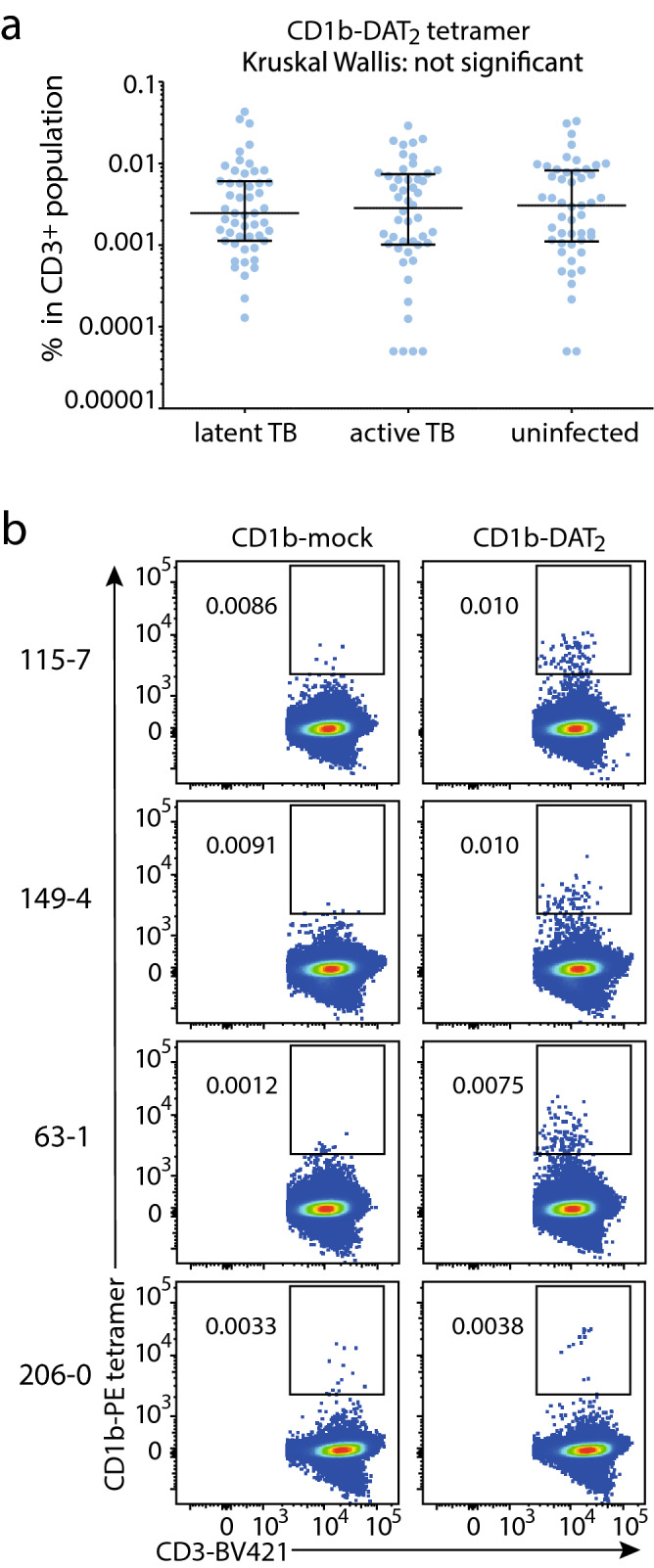
Quantification of CD1b-DAT_2_ tetramer binding T cells in a Peruvian TB cohort. (**A**) Frequencies of CD1b-mock and CD1b-DAT_2_ tetramer^+^ T cells among the 150 subjects of the Peruvian cohort, analyzed by TB disease status. Medians and interquartile ranges of tetramer^+^ T cells are depicted as a percent of total CD3^+^ cells. (**B**) Representative flow cytometry dot plots from subjects with CD1b-DAT_2_ tetramer positive T cells.

Next, we wanted to measure the frequency of DAT_2_-reactive T cells in a cohort of 150 human subjects^27,28^ to determine if there is an Mtb infection or disease-associated expansion of DAT-specific T cells. PBMCs were isolated from 50 Peruvian individuals with active tuberculosis (TB) before the start of anti-TB drug treatment. In addition, PBMCs were isolated from 50 patients with latent TB infection and 50 household contacts with Mtb exposure, but no documented infection (uninfected), based on IFN-γ release assay results^27,28^. To generate adequate numbers of T cells for the tetramer analysis, we expanded an aliquot of PBMCs by stimulation with anti-CD3 antibody and feeder cells, as previously described^28^. CD1b-synthetic DAT_2_ binding T cells were observed in subjects across all three groups, although in low frequencies. Comparing the median tetramer staining rate among the three groups based on TB disease status, the frequencies of CD1b-DAT_2_ tetramer positive T cells did not significantly differ among active TB patients, latently infected patients, and uninfected subjects as determined by the Kruskall-Wallis test (Fig. 4A). Staining patterns of expanded PBMCs with CD1b-synthetic DAT_2_ vary from broad smear of tetramer positive cells as illustrated by three subjects with tetramer-binding T cells (subjects 115-7, 149-4, and 63-1) to smaller high affinity populations, as seen for subject 206-0 (Fig. 4B). Thus, synthetic DAT_2_ is recognized by T cells in the blood, but frequencies of these cells did not increase after infection with Mtb.

## Discussion

Here we have characterized the antigenicity of DAT for the human immune system and show that synthetic DATs are able to act as both an innate and an adaptive agonist. Small differences in chemical structure between the three synthetic forms of DAT had strong effects on stimulation of innate versus adaptive receptors. We determined that DAT_3_, but not DAT_1_ or DAT_2_, behaved as a highly potent activator of the innate receptor Mincle, while DAT_1_ was by far the most potent compound recognized by polyclonal, ex-vivo T cells across multiple donors.

As predicated, DAT could be presented by CD1b and act as an antigen for T cells. Across multiple healthy donors we observed T cell binding to CD1b-DAT tetramers with frequencies similar to binding of CD1b-GMM and mycolic acid tetramers^28^. The highest percentage of CD1b-DAT tetramer^+^ cells was observed using synthetic DAT_1_ treated tetramers, followed by synthetic DAT_2_ treated CD1b tetramers, while the percentage CD1b-synthetic DAT_3_ tetramer^+^ T cells was extremely low. These results suggest that the composition of methyl-branched fatty acids of DAT strongly influences recognition by CD1b-reactive T cells.

Among the trehalose-based glycolipids that are made by Mtb, DAT is one of the smallest and simplest. Whereas sulfoglycolipids are sulfated on the 2′-position of the trehalose core and can carry up to four alkyl chains^9^, DAT is not sulfated and by definition carries two alkyl chains^16^. DAT carries an esterified unbranched saturated fatty acid on the 2-position of trehalose and a branched fatty acid on the 3-position: mycosanoic (DAT_1_), mycolipanolic (DAT_2_) or mycolipenic acid (DAT_3_)^16^. Thus, although sulfoglycolipids can carry longer and more complex branched fatty acids at the 2- and 3-position, Ac_2_SGL is the closest relative of DAT. The binding mechanism of Ac_2_SGL to CD1b is known and shows that CD1b presents Ac_2_SGL to T cells with the participation of endogenous spacer lipid that is simultaneously bound in the cleft^29^. The presence of these spacers in addition to Ac_2_SGL leads to rearrangement of the lipid-binding groove, allowing accommodation for bulky antigens. At the same time this rearrangement reduces the capacity of the A’ pocket of CD1b to accomodate the phthioceranoyl chain of Ac_2_SGL, forcing the first three methyl groups of the fatty acyl chain to remain exposed above the CD1b surface for recognition by TCR^29^. Since Ac_2_SGL and DAT show structural similarities, DAT might be presented by CD1b in a similar way, with the methyl branched motif exposed on the outer surface of CD1b. If that is true, differences in the exposed residues that are available for TCR recognition, such as the presence of the extra hydroxy group in the acyl chain of DAT_2_ and the α,β-unsaturation in DAT_3_, might explain the observed lack of cross-recognition by TCRs, such as HD1A. In addition, the differences in the number of C-methyl groups on the fatty acid of DAT_1_ (2 groups) and DAT_2_ and DAT_3_ (3 groups) could play a role in lack of cross-reactivity. However, the opposite effect was observed for Ac_2_SGL: an increased number of methyl-branched carbons led to an increase in the ability of the synthetic Ac_2_SGLs to stimulate T cells, which was true for up to four methyl groups^24^. Thus, the nature of the effect of methyl-branched fatty acids of DAT on T cell recognition by CD1b-reactive T cells can only be fully understood by additional analyses, including protein crystallography of the trimolecular complex of CD1b-DAT-TCR.

The Peruvian TB cohort data shows that synthetic DAT_2_ is recognized by T cells in people with active TB, healthy latently Mtb-infected, and uninfected controls. However, a difference in frequencies of CD1b-DAT binding T cells among highly exposed groups that differed in their IGRA status was not observed, similar to other CD1 tetramer studies in cohorts^28,30^. Also, the range of percentage of CD1b-DAT_2_ tetramer^+^ T cells of Peruvian subjects was similar to the Boston healthy donors. Together, these data suggest that CD1b-DAT_2_-specific T cells do not expand upon Mtb exposure, or, if they do, it is not detectable among T cells that circulate in the blood. Recent studies have suggested that total blood MR1-reactive T cells can stay unchanged or fall in the setting of infection or antigen-stimulation^27,31–33^. Thus, a more general perspective to emerge from these studies is that blood-based quantification is not a reliable measure of total body T cell dynamics. Nevertheless, these studies provide proof of principle for DAT specific T cells response and point to DAT_1_ as the T cell antigen with highest response.

In conclusion, our results show that the mycobacterial lipid DAT is an antigen for T cells as well as a stimulating ligand for the Mincle receptor, but the structural differences in the fatty acyl chains of the different forms of DAT strongly influence the type of biological response they elicit.

## Material and methods

### HPLC–MS of synthetic DAT

Diacyl trehaloses were synthesized as previously published (13). The synthetic DAT compounds were analyzed on an Agilent Technologies 6530 Accurate-Mass Q-TOF HPLC–MS system. Reversed phase liquid chromatography (LC) used a C-18 HPLC column (Agilent Poroshell 120, 2.7 mm, 4.6 mm × 100 mm) and a gradient method with 7:3 methanol:water (solvent A) and 85:15 1-propanol:cyclohexane (solvent B). Both solvents contained 2.0 mM ammonium formate. 0.1% water was added to solvent B to aid dissolution. The solvent gradient used a 0.5 mL/min flowrate throughout and started at 60% solvent B, increased linearly starting at 1.0 min and ending at 100% solvent B at 10.0 min, and finally holding at 100% B until 15.0 min. Runs were initiated with 10 µL injections of the synthetic compounds at a concentration of 20 ng/mL in starting mobile phase. Detected ions were analyzed using Agilent Technologies MassHunter Qualitative Analysis B.07.00 software.

### Mincle activation assay

For the cellular Mincle assays DAT_1_, DAT_2_, and DAT_3_ were dissolved in chloroform/methanol at 1 mg/mL, diluted in isopropanol to appropriate concentrations and added to a 96-well plate at 20 µL/well. After evaporation of the isopropanol, 30,000 2B4-NFAT-GFP reporter cells expressing mouse Mincle were added to each well in 100 µL medium. After incubation at 37 °C for 24 h the expression of NFAT-GFP was analyzed by flow cytometry.

### Human subjects

The Brigham and Women's Hospital Specimen Bank, Boston, provided de-identified leukoreduction filters from local blood bank donors for PBMC isolation. Subjects with pulmonary TB disease and their household contacts were recruited through Socios En Salud, an affiliate of Partners in Health, based in Lima, Peru^27,28^. We enrolled 50 patients with culture confirmed pulmonary TB and 100 of their asymptomatic household contacts of whom 50 had positive IGRA tests, as determined by the QuantiFERON TB-Gold In-Tube assay (Qiagen) and 50 subjects were IGRA negative (classified as “exposed but uninfected”). Participants were at least 14 years old and had a negative HIV serology test. Peripheral blood mononuclear cells (PBMC) were isolated from 50 mL of blood and cryopreserved at 5 × 10^6^ cells per aliquot. The Institutional Review Board of the Harvard Faculty of Medicine and Partners Healthcare, and the Institutional Committee of Ethics in Research of the Peruvian Institutes of Health approved this study protocol. All adult study participants and parents or legal guardians of minors had to provide informed consent, while minors provided assent. All methods were performed in accordance with the relevant guidelines and regulations.

### Tetramers

For lipid loading, WT CD1b monomers were obtained from the NIH tetramer facility. In a 10 mm wide glass tube, 16 μg of dry lipid was sonicated at 37 °C for 1 h in 45 μL of 0.5% CHAPS 50 mM sodium citrate buffer pH 4.5 for DAT-treated tetramers, as previously described for other lipid ligands^28^. For CD1b-mock no lipid was added to the tube. Subsequently, CD1b monomers (10 μg) were added to the tubes and incubated overnight at 37 °C. The next day the solution of the monomers was neutralized to pH 7.4 by adding 5 μL 1 M Tris pH 8. Monomers were tetramerized using streptavidin-APC (Molecular Probes) or streptavidin-PE (Invitrogen).

### T cell lines and T cell assays

For generation of T cell lines, total PBMC or PBMC-derived T cells were stained with CD1b-DAT tetramer and anti-CD3. PBMCs were sorted for double positive staining of CD3 and tetramer. Expansion of sorted cells was performed by plating cells at 100–700 cells/well in round-bottom 96-well plates containing 2.5 × 10^5^ irradiated allogeneic PBMCs, 5 × 10^4^ irradiated Epstein Barr Virus transformed B cells, and 30 ng/mL anti-CD3 antibody (clone OKT3) per plate as described previously^28^. The next day human IL-2 was added to the wells. After 2 weeks, sorting and expansion procedure was repeated as needed. For ELISPOT assays, cocultures of 4 × 10^4^ APCs (G4 monocytes) pre-incubated with DAT for 30 min 37 °C and 1 × 10^3^ T cells were incubated for 16 h in a Multiscreen-IP filter plate (96 wells; Millipore) coated according to the manufacturer’s instructions (Mabtech). For blocking, APCs were preincubated for 1 h at 37 °C with anti-CD1b blocking antibody BCD1b3.1 or control IgG P3 (10 μg/mL) before adding T cells.

### Staining protocol

T cells were enriched by depletion of non-T cells using the Pan T cell Isolation Kit (Miltenyi Biotec) according to manufacturer’s protocol. Human enriched T cells and T-cell lines were stained with tetramers at 2 μg/mL in PBS containing 1% BSA and 0.01% sodium azide. Cells and tetramer were incubated for 10 min at room temperature in the dark, followed by addition of cell surface antibodies for 10 min at room temperature as described previously^27,28^. Subsequently, cells were treated with unlabeled OKT3 antibody and incubated for 20 min at 4 °C. Cells were analyzed using the BD LSRFortessa flow cytometer and FlowJo software. For staining of PBMCs from Peruvian participants ~ 3 × 10^6^ cells were stained with a “live-dead” fixable blue cell stain (Molecular Probes), then treated with tetramer in for 10 min at room temperature, followed by cell surface antibodies for 5 min. Subsequently, cells were treated with unlabeled OKT3 antibody and incubated for 5 min at room temperature, followed by 10 min at 4 °C. Cells were fixed in fresh 2% paraformaldehyde (Electron Microscopy Sciences) in PBS for 20 min. Antibodies that were used: CD3-BV421 (UCHT1; Biolegend), CD3-FITC (SK7; BD Bioscience).

### TCR sequencing

TCR sequences were determined by isolating RNA from bulk sorted T cell populations using the RNeasy kit (QIAGEN), followed by complementary DNA synthesis using the QuantiTect Reverse Transcription Kit (QIAGEN). TCR transcripts were amplified using a multiplex approach^12^, followed by direct Sanger sequencing of the PCR product.

## Supplementary Information

Supplementary Information
