# Supplementary material for: Synthetic mycobacterial diacyl trehaloses reveal differential recognition by human T cell receptors and the C-type lectin Mincle
Source: Sci Rep. 2021 Jan 21;11:2010. doi: 10.1038/s41598-021-81474-3 (PMC7820438; doi:10.1038/s41598-021-81474-3)
Supplement: Supplementary file 1 — Supplementary Information [file 41598_2021_81474_MOESM1_ESM.pdf]

Supplementary information for:

Synthetic mycobacterial diacyl trehaloses reveal differential recognition by human T cell receptors and the C-type lectin Mincle

Josephine F. Reijneveld[1,2,3], Mira Holzheimer[3], David C. Young[1], Kattya Lopez[1,4], Sara Suliman[1], Judith Jimenez[4], Roger Calderon[4], Leonid Lecca[4], Megan B. Murray[5], Eri Ishikawa[6,7], Sho Yamasaki[6,7], Adriaan J. Minnaard[3], D. Branch Moody[1], Ildiko Van Rhijn*[1,2]

1. Division of Rheumatology, Inflammation, and Immunity, Brigham and Women's Hospital and Harvard Medical School, Boston, MA, United States
2. Department of Infectious Diseases and Immunology, Faculty of Veterinary Medicine, Utrecht University, Utrecht, Netherlands
3. Stratingh Institute for Chemistry, University of Groningen, Groningen, The Netherlands
4. Socios En Salud, Lima, Peru
5. Division of Global Health Equity, Department of Global Health and Social Medicine, Brigham and Women's Hospital and Harvard Medical School, Boston, MA, United States
6. Department of Molecular Immunology, Research Institute for Microbial Diseases, Osaka University, Suita, Osaka, Japan.
7. Laboratory of Molecular Immunology, Immunology Frontier Research Center, Osaka University, Suita, Osaka, Japan

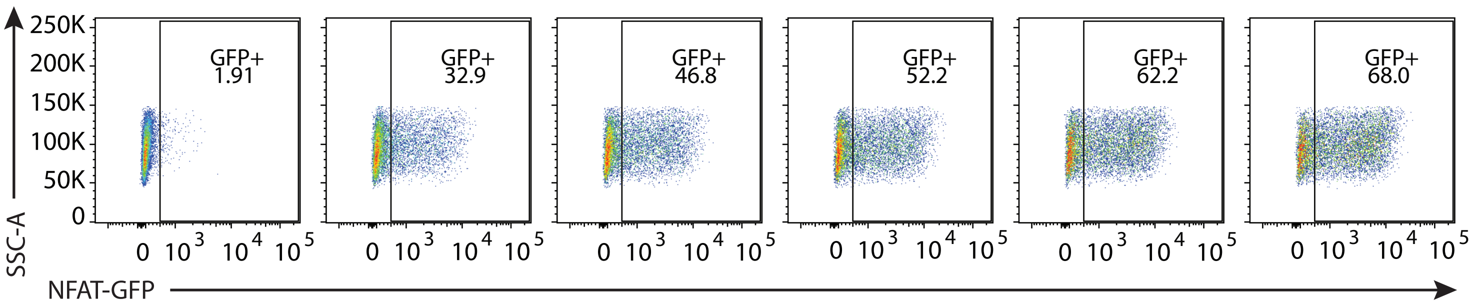

Figure S1. Gating strategy for Figure 1d. NFAT-GFP expression in reporter cells expressing murine Mincle and it signaling subunit FcεRγ chain, after stimulation with DAT_3.

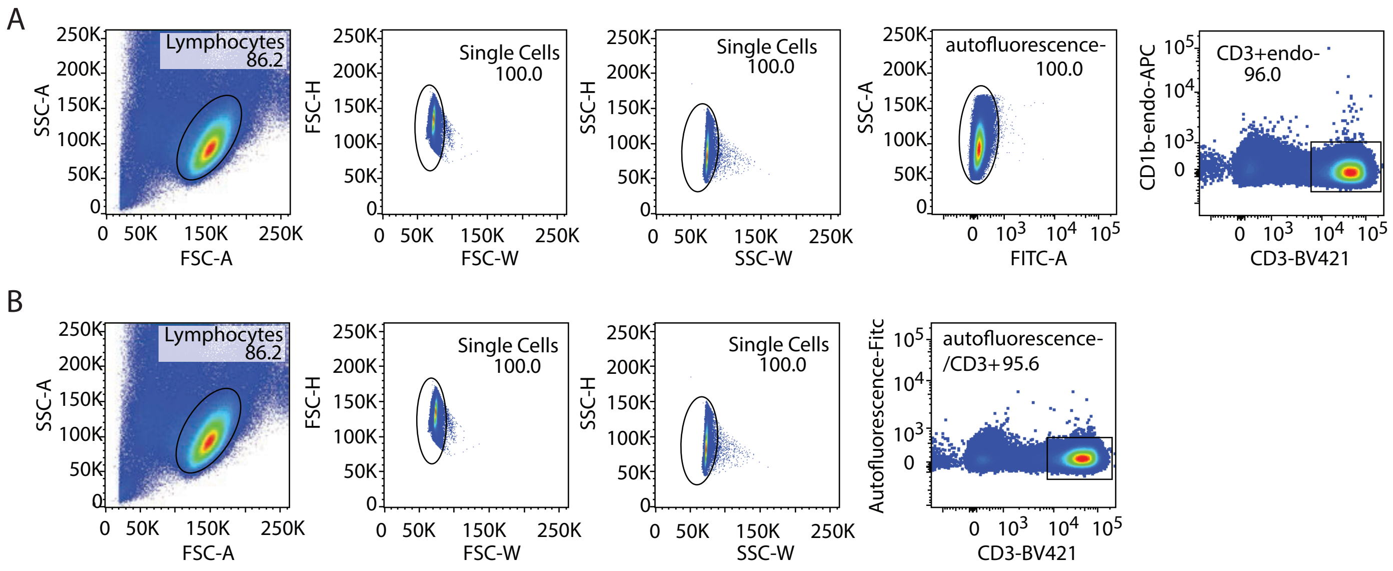

Figure S2. Gating strategy of T cells enriched from PBMC by column purification from healthy donors. (A) Pre-gating on $CD3^+CD1b\text{-}endo^-$ T cells. (B) Pre-gating on $CD3^+$ cells.

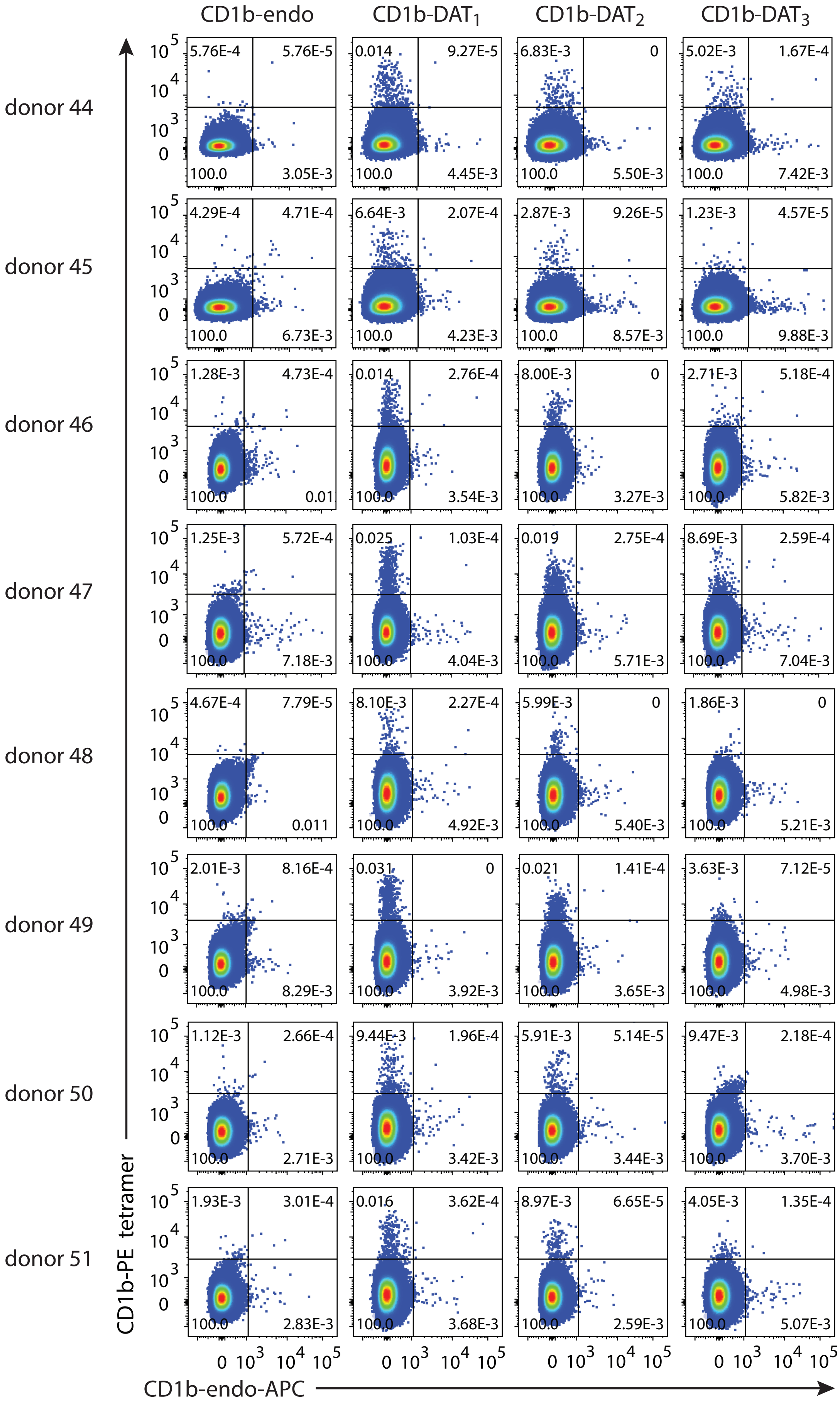

Figure S3. Flow cytometry dot plots of T cells enriched from PBMC by column purification from 8 healthy donors stained with CD1b-endo, -DAT_1, -DAT_2, and -DAT_3 tetramers after pre-gating on $CD3^+$ T cells.

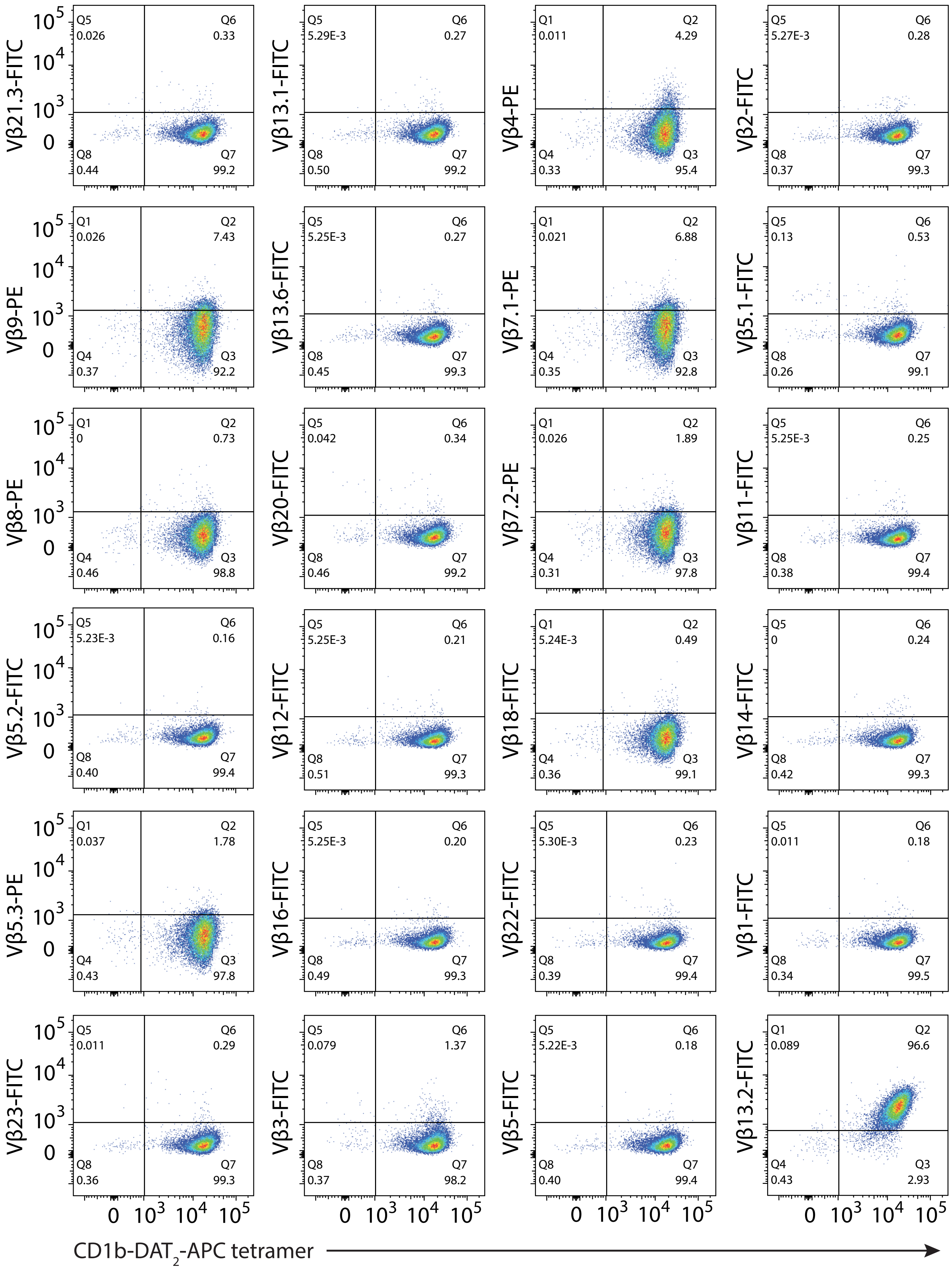

Figure S4. Flow cytometry dot plots of line HD1A stained with CD1b-DAT$_2$ tetramer and 24 different anti-Vβ antibodies after pre-gating for CD3$^+$ T cells.
